# Dietary fat intake and ovarian cancer risk: a meta-analysis of epidemiological studies

**DOI:** 10.18632/oncotarget.8940

**Published:** 2016-04-22

**Authors:** Wenlong Qiu, Heng Lu, Yana Qi, Xiuwen Wang

**Affiliations:** ^1^ Department of Oncology, Qilu Hospital, School of Medicine, Shandong University, Jinan, Shandong, China

**Keywords:** meta-analysis, ovarian epithelial carcinoma, dietary fat

## Abstract

Observational studies assessing the association of dietary fat and risk of ovarian cancer yield discrepant results. Pertinent prospective cohort studies were identified by a PubMed search from inception to December 2015. Sixteen independent case-control and nine cohort studies on dietary fat intake were included, with approximately 900,000 subjects in total. Relative risks (RRs) with 95% confidence intervals were pooled using a random effects model. Heterogeneity, sensitivity analysis and publication bias were assessed; subgroup analysis and analysis stratified by EOC histology were conducted. The reported studies showed a significant increase of ovarian cancer risk with high consumption of total-, saturated-, and trans-fats, while serous ovarian cancer was more susceptible to dietary fat consumption than other pathological subtypes. No evidence of positive association between dietary fat intake and ovarian cancer risk was provided by cohort studies. Menopausal status, hormone replacement therapy, body mass index (BMI), and pregnancy times, modified the objective associations. In conclusion, the meta-analysis findings indicate that high consumption of total, saturated and trans-fats increase ovarian cancer risk, and different histological subtypes have different susceptibility to dietary fat.

## INTRODUCTION

Ovarian cancer is considered the sixth most commonly diagnosed cancer among women and the second cause of gynecologic cancer mortality worldwide [1, 2]. The prognosis of ovarian cancer is poor, with the initial diagnosis in most patients made at an advanced stage [3, 4]. The noticeable relationship between ovarian cancer incidence and geographical regions suggested that dietary habits and ethnic variations are potentially modifiable factors [5], whose etiologic role in ovarian cancer risk, however, remains undefined [6].

Dietary fat, as one of the most controversial dietary factors in nutritional epidemiology, has been reported with positive correlations with breast [7] and gastric [8] cancers in two recent meta-analyses, and elevated ovarian cancer risk in early ecologic studies [5, 9]. Although multiple epidemiologic studies have explored the associations between dietary fat consumption and risk of ovarian cancer, no definite conclusion have been drawn, and the dietary fat varieties as well as pathological types of ovarian cancer increase the complexity of this research topic. The results of two meta-analyses [10, 11] and a pooled analysis [12] that included data from 12 cohort studies also reached inconsistent conclusions. Therefore, we conducted a meta-analysis of case-control and cohort studies with more-detailed analyses of 1) the epidemiologic evidence regarding the association of dietary fat consumption with risk of ovarian cancer, 2) the association between dietary fat intake and the risk of ovarian cancer and pathological subtypes. This analysis was based on dietary fat types, and we extended the previous analyses [10, 11] with more included studies and dietary fat types, and an assessment stratified by EOC histology.

## RESULTS

**Figure 1:**
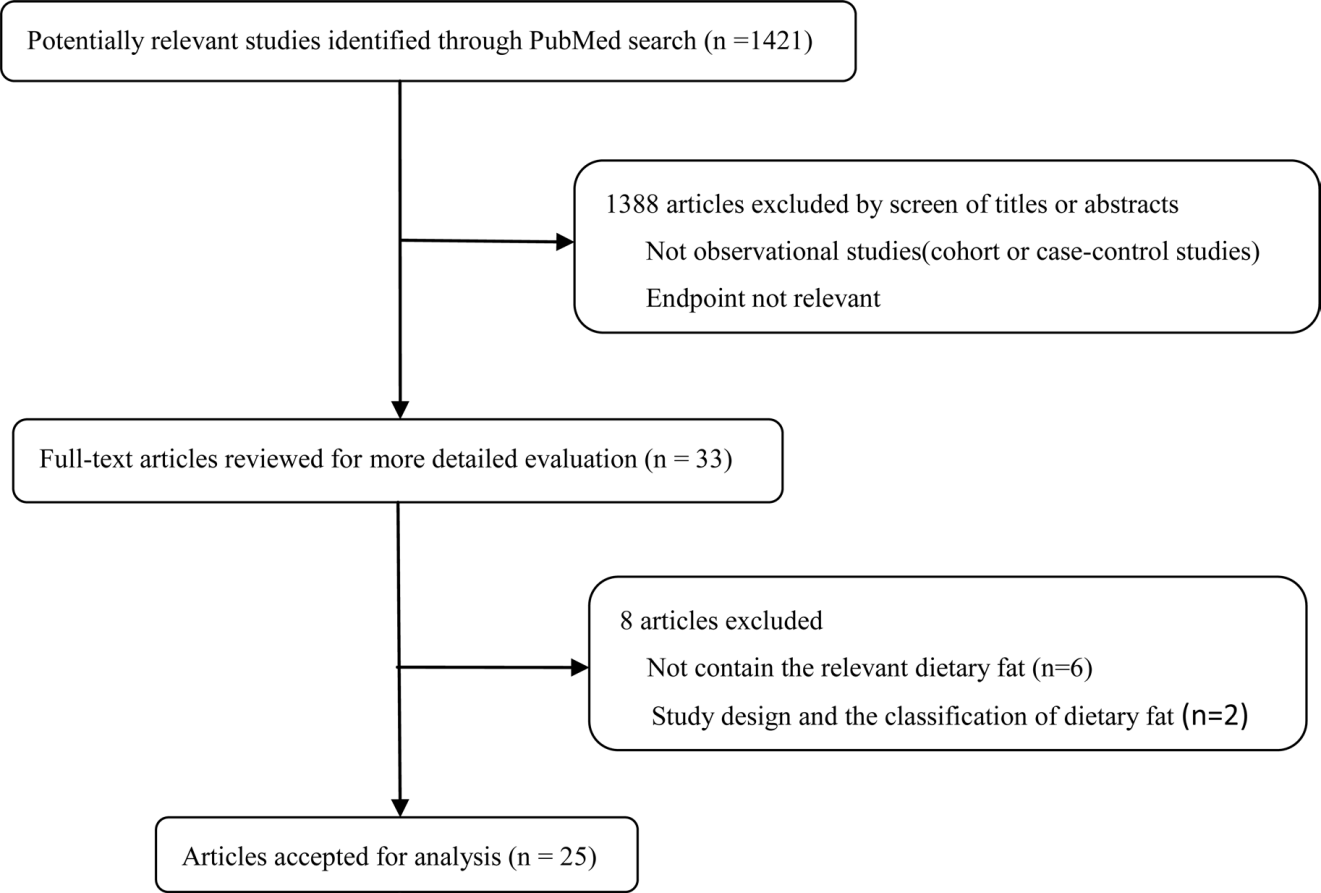
Flow chart

We found 1421 publications from the electronic and manual literature searches. Thirty-three potentially relevant publications [16–31] [32–47] [48] appeared to meet the specified protocol inclusion criteria after initial screening. Through further reading, six publications [41–46] contained no relevant dietary fat; two publications [47, 48] were excluded for design and dietary fat classification. Two American publications [17, 18] assessed the same study population, and eligible data were extracted from both; two Chinese publications were treated likewise [25, 28] (Figure 1).

Finally, Twenty-five publications [16–31] [32–40], including sixteen case-control [16–31] (Table 1) and nine cohort [32–40] (Table 2) studies were included in the analysis of dietary fat intake and ovarian cancer risk. Results were separated by dietary fat types, including total, saturated, monounsaturated, polyunsaturated, animal, plant, dairy fat, and trans-fats.

**Table 1:** The characteristics and relative risks [RRs; 95% confidence interval (CI)] for case-control studies on dietary fat and ovarian cancer

Author(Country)	Publication yr	Case/Control	Type of Fat	RR (95% CI)	Adjusted confounding factors
Cramer[16] (America)	1984 (1978-1981)	215/215	Animal fat	1.83 (1.00-3.38)	Weight/height^2^
Risch[17, 18] (Canada)	1994 (1989-1992)	450/564	Total fat Saturated fat Monounsaturated fat Polyunsaturated fat	1.16 (0.86-1.57) 1.23 (0.97-1.58) 1.07 (0.90-1.27) 0.86 (0.69-1.07)	Age, total calorie intake, no. of full-term pregnancies, duration of OC use
Shu[19] (China)	1989 (1984-1986)	172/172	Total fat Animal fat Plant fat	1.9 (1.2-4.4) 1.7 (1.0-3.2) 0.8 (0.4-1.4)	income, no. of live births, history of ovarian cysts, smoking history, OC use, IUD use, tubal ligation
Slattery[20] (America)	1989 (1984-1987)	85/492	Total fat Saturated fat Monounsaturated fat Polyunsaturated fat	1.3 (0.7-2.3) 1.3 (0.6-2.6) 1.3 (0.7-2.3) 1.2 (0.6-2.3)	Age, BMI, no. of pregnancies
Tzonou[21] (Greece)	1993 (1989-1991)	189/200	Total fat Saturated fat Monounsaturated fat Polyunsaturated fat	0.97 (0.76-1.24) 1.17 (0.88-1.55) 0.86 (0.71-1.05) 0.95 (0.77-1.17)	Age, years of schooling, parity, age at 1st birth, menopausal status, energy intake
La Vecchia[22] (Italy)	1987 (1983-1986)	455/1385	Total fat	2.14 (1.59-2.88)	Age, interviewer, marital status, social class, education, parity, age at 1st birth, age at menarche, menopausal
Webb[23] (Australia)	1998 (1990-1993)	824/1132	Total fat	1.86 (1.03-3.37)	Age, education, BMI, smoking, parity, OC use, total energy intake
Pan[24] (Canada)	2004 (1994-1997)	442/2135	Total fat Saturated fat Monounsaturated fat Polyunsaturated fat	1.21 (0.88-1.65) 1.06 (0.78-1.45) 1.26 (0.92-1.72) 1.28 (0.94-1.76)	Age, residence, education, alcohol consumption, smoking, BMI, caloric intake, recreational physical activity, number of live births, menstruation years, and menopause status
Zhang[25] (China)	2002 (1999-2000)	254/652	Animal fat Plant fat	4.55 (2.2-9.3) 1.03 (0.6-1.9)	Age, residence, education, alcohol consumption, smoking, BMI, tube ligation, menopause status
McCann[26] (America)	2003 (1986-1991)	124/696	Total fat Saturated fatty acid Monounsaturated fat Polyunsaturated fat	1.51 (0.57–4.02) 1.46 (0.68–3.15) 1.77 (0.73–4.31) 0.63 (0.28–1.41)	Age, education, total months menstruating, difficulty becoming pregnant, OC use, menopausal status and total energy intake
Merritt[27] (America)	2014 (1992-2008)	1872/1978	Total fat Animal fat Dairy fat Saturated fat Plant fat Trans fat Monounsaturated fat Polyunsaturated fat	1.07 (0.89-1.29) 1.04 (0.87-1.26) 0.95 (0.79-1.14) 1.11 (0.92-1.34) 0.98 (0.81-1.17) 1.30 (1.08-1.57) 0.97 (0.81-1.18) 0.82 (0.68-0.99)	Age, study centre (MA, NH), study phase, number of pregnancies, OC use, family history of ovarian cancer tubal ligation
Zhang[28] (China)	2003 (1999-2000)	254/652	Total fat	2.17 (1.26-3.75)	Age, locality, education, family income, BMI, total energy intake, tobacco smoking, alcohol, parity, menopausal status, OC use
Salazar-Martinez[29] (Mexico)	2002 (1995-1997)	84/629	Total fat Saturated fat Monounsaturated fat Polyunsaturated fat Animal fat Plant fat	0.60 (0.33-1.06) 0.56 (0.31-1.02) 0.54 (0.30-0.99) 0.61 (0.34-1.11) 0.66 (0.37-1.19) 0.81 (0.46-1.45)	Age, weight change, total energy intake, number of live births, physical activity, diabetes
Chiaffarino[30] (Italy)	2007 (1992-1999)	750/2411	Monounsaturated fat Polyunsaturated fat	0.80 (0.66-0.96) 0.96 (0.76-1.21)	education, parity, oral contraceptive use, family history of ovarian and/or breast cancer in first degree relatives
Hu[31] (Canada)	2011 (1994-1997)	442/5039	Trans fat	1.04 (0.68-1.58)	Age, province, education, BMI, alcohol drinking, pack-year smoking, total of vegetable and fruit intake, monounsaturated fat, polyunsaturated fat, total energy intake, number of live births and years of menstruation

**Table 2:** The characteristics and relative risks [RRs; 95% confidence interval (CI)] for cohort studies on dietary fat and ovarian cancer

Author (Country)	Publication yr	Case/Total	Type of Fat	RR (95% CI)	Adjusted confounding factors
Merritt[32] (America)	2014 (1980-2009)	764/95452	Dairy fat	1.01 (0.80-1.27)	Caloric intake, number of pregnancies, parity, OC use, menopausal status, tubal ligation, family history
Bertone[33] (America)	2002 (1980-1996)	301/80258	Total fat Animal fat Dairy fat Saturated fat Plant fat Monounsaturated fat Polyunsaturated fat Trans fat	1.03 (0.72-1.45) 0.95 (0.66-1.38) 1.06 (0.73-1.54) 0.91 (0.62-1.32) 0.98 (0.68-1.43) 1.07 (0.75-1.52) 1.14 (0.79-1.63) 1.03 (0.72-1.47)	Age, parity, age at menarche, OC use, menopausal status, postmenopausal hormone use, tubal ligation, smoking status
Merritt[34] (Europe)	2014	1095/325007	Total fat Animal fat Plant fat Saturated fat Monounsaturated fat Polyunsaturated fat	1.16 (0.96-1.40) 0.96 (0.80-1.15) 1.22 (0.98-1.52) 1.17 (0.97-1.40) 1.16 (0.93-1.44) 1.22 (1.02-1.48)	OC use, number of children, menopausal status at enrolment, total energy intake
Blank[35] (America)	2012 (1995-2005)	695/151552	Total fat Animal fat Plant fat Saturated fat Monounsaturated fat Polyunsaturated fat	1.28 (1.01-1.63) 1.30 (1.02-1.66) 1.00 (0.79-1.27) 1.03 (0.71-1.50) 1.01 (0.63-1.60) 1.28 (0.92-1.77)	Age, race, education, BMI, family history, OC use, parity, menopausal hormone therapy use, total energy intake
Kiani[36] (America)	2006 (1976-1992)	71/ 13,281	Dairy fat	0.94 (0.70-1.27)	Age, parity and BMI, and also for age at menopause and hormone replacement therapy in postmenopausal analyses
Mommers[37] (Netherlands)	2006 (1986-1997)	252/2216	Dairy fat	1.53 (1.00-2.36)	Age, height, smoking, number of cigarettes smoked daily, OC use and parity, and dairy products
Kushi[38] (America)	1999 (1986-1995)	139/ 29,083	Total fat Animal fat Saturated fat Plant fat Monounsaturated fat Polyunsaturated fat	0.80 (0.47-1.36) 0.98 (0.57-1.69) 1.17 (0.69-1.97) 0.75 (0.44-1.27) 0.65 (0.38-1.13) 0.63 (0.38-1.03)	Age, total energy intake, no. of live births, age at menopause, family history of ovarian cancer, hysterectomy, waist-to-hip ratio, level of physical activity, smoking, education
Chang[39] (America)	2007 (1995-2003)	280/97275	Total fat Saturated fat	0.85 (0.58-1.24) 0.72 (0.48-1.08)	Race, energy intake, parity, OC use, exercise, wine consumption, menopausal status, hormone therapy
Gilsing[40] (Netherlands)	2011 (1986-2002)	340/ 62,573	Total fat Animal fat Plant fat Dairy fat Saturated fat Monounsaturated fat Polyunsaturated fat Trans fat	1.04 (0.73-1.49) 1.30 (0.93-1.83) 0.64 (0.45-0.91) 1.28 (0.91-1.80) 1.48 (0.94-2.34) 0.90 (0.55-1.46) 0.89 (0.47-1.01) 1.51 (1.04-2.20)	Age, total energy intake, parity (number of children), and use of oral contraceptives use

### Total fat

**Figure 2:**
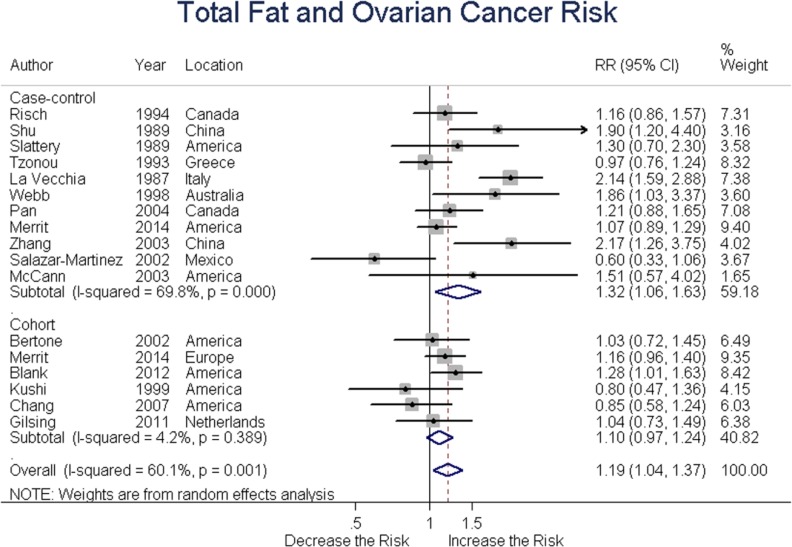
Relationship between total fat intake and ovarian cancer risk

Eleven case-control and six cohort studies assessed total fat intake and ovarian cancer risk. Summary RR was 1.32 (95% CI = 1.06-1.63, *P* = 0.017) for case-control and 1.10 (95% CI = 0.97-1.24, *P* = 0.25) for cohort studies, with an overall RR of 1.19 (95% CI = 1.04-1.37, *P* = 0.015) for all studies. These results suggested a positive association between total fat intake and ovarian cancer risk. (Figure 2)

### Animal fat

**Figure 3:**
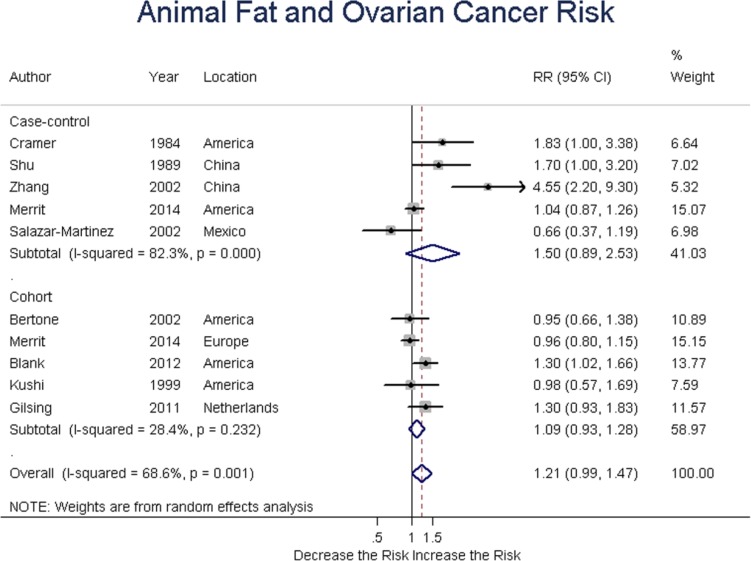
Relationship between animal fat intake and ovarian cancer risk

Five case-control and five cohort studies analyzed animal fat intake and ovarian cancer risk. Summary RR was 1.50 (95% CI = 0.89-2.53, *P* = 0.125) for case-control, and 1.09 (95% CI = 0.93-1.28, *P* = 0.272) for cohort studies; the overall RR was 1.21 (95% CI = 0.99-1.47, *P* = 0.065) for all studies. Taken together, these results suggested no association between animal fat intake and ovarian cancer risk. (Figure 3)

### Plant fat

**Figure 4:**
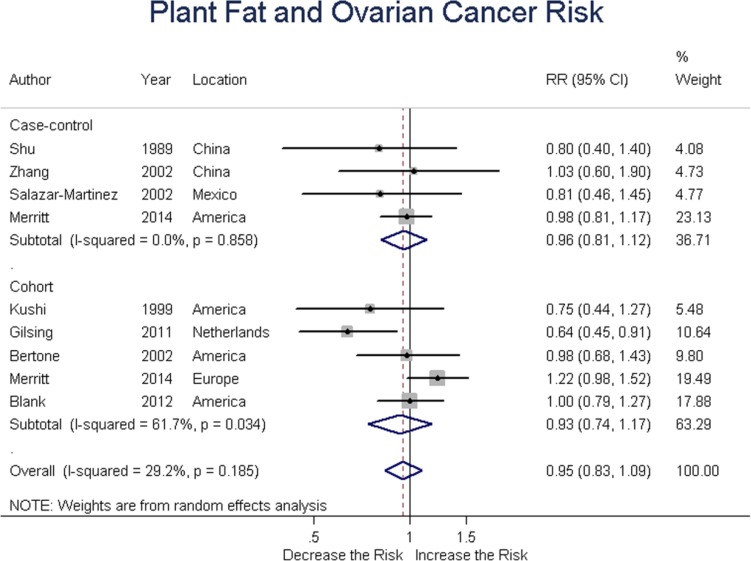
Relationship between plant fat intake and ovarian cancer risk

Four case-control and five cohort studies evaluated plant fat intake and ovarian cancer risk. Summary RR was 0.96 (95% CI = 0.81-1.12, *P* = 0.586) for case-control, and 0.93 (95% CI = 0.74-1.17, *P* = 0.053) for cohort studies, with an overall RR of 0.95 (95% CI = 0.83-1.09, *P* = 0.472). These results suggested no association between plant fat intake and ovarian cancer risk. (Figure 4)

### Saturated fat

**Figure 5:**
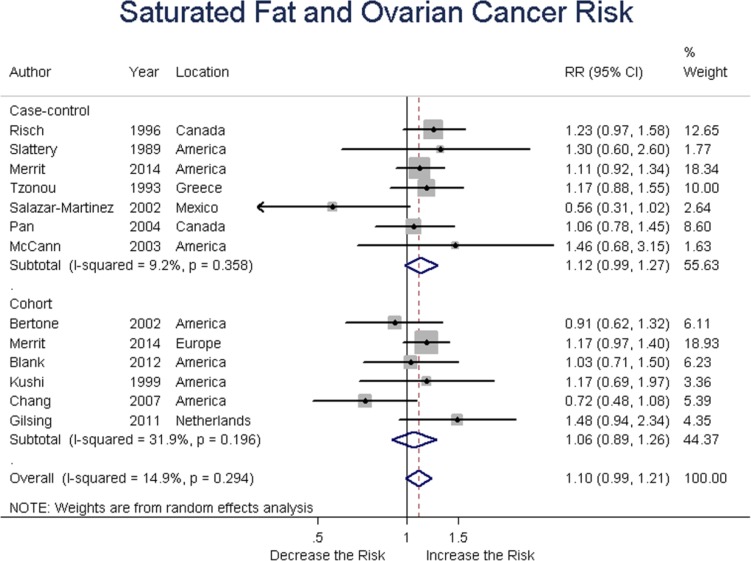
Relationship between saturated fat intake and ovarian cancer risk

Seven case-control and six cohort studies assessed saturated fat intake and ovarian risk. Summary RR was 1.11 (95% CI = 0.98-1.27, *P* = 0.147) for case-control, and 1.06 (95% CI = 0.89-1.26, *P* = 0.0.521) for cohort studies; the overall RR was 1.09 (95% CI = 0.98-1.21, *P* = 0.103). After exclusion of 1 study [29] for small bias and sensitivity data, the results changed as follows, respectively, 1.15 (95% CI = 1.02-1.30, *P* = 0.026), 1.06 (95% CI = 0.89-1.26, *P* = 0.521), 1.12 (95%CI = 1.02-1.22, *P* = 0.014), without heterogeneity (Q = 8.54, *P* = 0.577, I2 = 0.0%) between studies. These results suggested a positive association between saturated fat intake and ovarian cancer risk. (Figure 5)

### Monounsaturated fat

**Figure 6:**
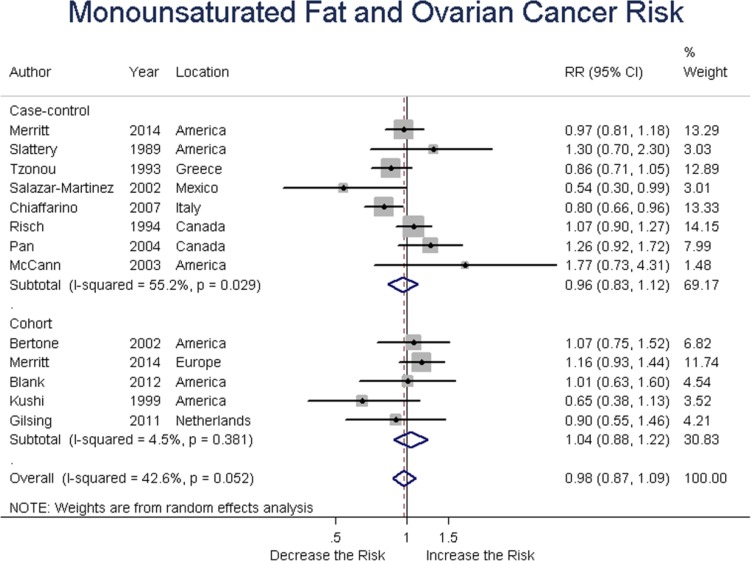
Relationship between monounsaturated fat intake and ovarian cancer risk

Eight case-control and five cohort studies analyzed monounsaturated fat intake and ovarian cancer risk. Summary RR was 0.96 (95% CI = 0.83-1.12, *P* = 0.477) for case-control, and 1.04 (95% CI = 0.88-1.22, *P* = 0.649) for cohort studies; the overall RR was 0.98 (95% CI = 0.87-1.09, *P* = 0.556) for all studies. These results suggested no association between total fat intake and ovarian cancer risk. (Figure 6)

### Polyunsaturated fat

**Figure 7:**
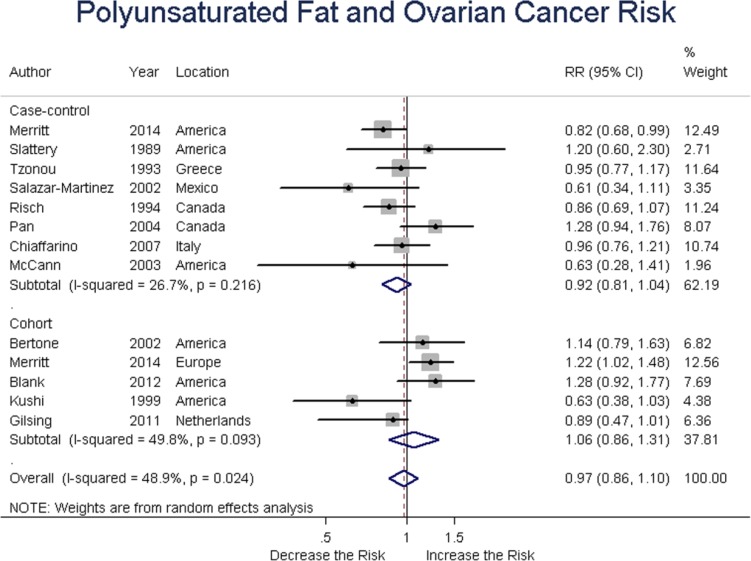
Relationship between polyunsaturated fat intake and ovarian cancer risk

Eight case-control and five cohort studies involved polyunsaturated fat intake and ovarian cancer risk. Summary RR was 0.92 (95% CI = 0.81-1.04, *P* = 0.223) for case-control, and 1.06 (95% CI = 0.86-1.31, *P* = 0.570) for cohort studies, with an overall RR of 0.97 (95% CI = 0.86-1.10, *P* = 0.760) for all studies. These findings suggested no association between polyunsaturated fat intake and ovarian cancer risk. (Figure 7)

### Dairy fat

**Figure 8:**
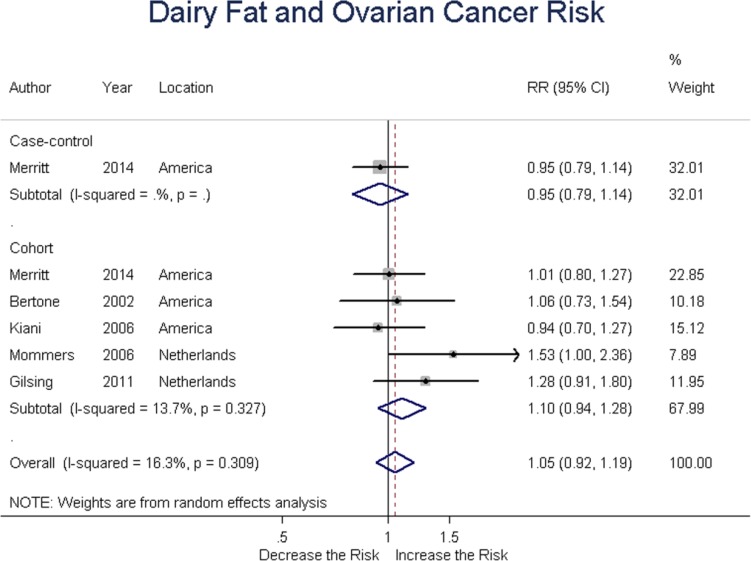
Relationship between dairy fat intake and ovarian cancer risk

One case-control and five cohort studies assessed dairy fat intake and the risk of ovarian cancer. Summary RR was 1.10 (95% CI = 0.94-1.28, *P* = 0.242) for cohort studies, with an overall RR of 1.05 (95% CI = 0.92-1.19, *P* = 0.478) for all studies. These results suggested no association between dairy fat intake and ovarian cancer risk. (Figure 8)

### Trans-fat

**Figure 9:**
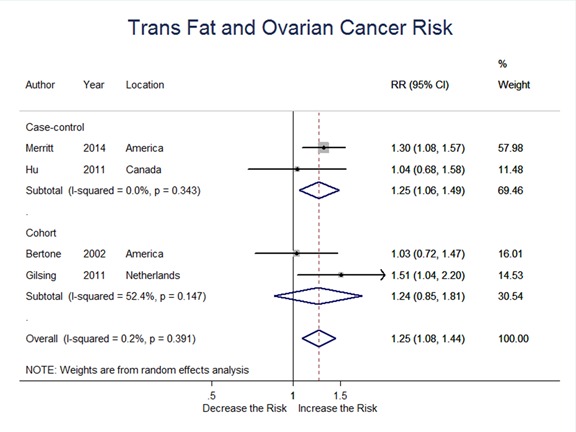
Relationship between trans fat intake and ovarian cancer risk

Two case-control and two cohort studies evaluated trans-fat intake and ovarian cancer risk. Summary RR was 1.25 (95%CI = 1.06-1.49, *P* = 0.010) for case-control, and 1.24 (95% CI = 0.85-1.81, *P* = 0.285) for cohort studies; the overall RR was 1.25 (95% CI = 1.08-1.44, *P* = 0.002) for all studies. These results suggested a significant positive association between trans-fat intake and ovarian cancer risk. (Figure 9)

### Dietary fat consumption and ovarian cancer subtypes

Five studies involved dietary fat intake and risk of ovarian cancer subtypes. Significant positive association was found between total fat, saturated fat, trans-fat intake and serous ovarian tumor risk. High saturated fat intake was associated with a 34% increase in endometroid ovarian cancer risk. The RR for high animal fat intake was 1.36 (95% CI = 1.08-1.73, *P* = 0.011), suggesting a significant positive association between animal fat consumption and mucinous ovarian cancer risk. (Table 3)

**Table 3:** Relative risks (RR) of ovarian cancer and corresponding 95% confidence intervals (CI) according to histological types and intake of dietary fat

Types of dietary fat	Histological types							
	Serous tumor		Endometroid tumor		Mucinous tumor		Other tumor	
	RR (95%CI)	*P*	RR (95%CI)	*P*	RR (95%CI)	*P*	RR (95%CI)	*P*
Total fat	1.12(1.01,1.24)	0.025	1.05(0.85,1.30)	0.628	1.15(0.69,1.90)	0.591	1.17(0.69,1.99)	0.561
Animal fat	1.07(1.00,1.16)	0.061	0.96(0.79,1.17)	0.715	1.36(1.08,1.73)	0.011	0.82(0.49,1.38)	0.456
Plant fat	1.07(0.95,1.21)	0.278	0.92(0.76,1.11)	0.391	0.96(0.73,1.27)	0.778	1.22(0.71,2.09)	0.468
Saturated fat	1.26(1.05,1.52)	0.012	1.34(1.08,1.66)	0.008	1.29(0.93,1.81)	0.132	1.19(0.40,3.51)	0.754
Monounsaturated fat	0.97(0.82,1.14)	0.692	0.78(0.57,1.06)	0.115	1.09(0.88,1.35)	0.428	0.84(0.58,1.21)	0.350
Polyunsaturated fat	0.97(0.78,1.21)	0.778	1.07(0.86,1.32)	0.564	1.15(0.93,1.43)	0.203	0.99(0.61,1.62)	0.974
Dairy fat	0.89(0.72,1.10)	0.283	1.13(0.79,1.61)	0.501	1.17(0.62,2.22)	0.631	0.75(0.44,1.27)	0.287
Trans fat	1.26(1.02,1.61)	0.044	1.27(0.89,1.81)	0.466	1.33(0.72,2.44)	0.358	1.10(0.64,1.89)	0.729

*P*-value for significant test. (*P* < 0.050)

Abbreviations: RR: relative risk, CI: confidence interval

### Subgroup and sensitivity analysis

Subgroup analysis stratified by the geographic areas, study types and confounding factors of included studies was performed. Saturated fat (RR = 1.20, 95% CI = 1.04-1.39) and dairy fat (RR = 1.37, 95% CI = 1.05, 1.79) intake could increase ovarian cancer risk in European populations; a positive trend was present among American populations, while an inverse trend was found in other populations (including Asians and South Americans). The conclusions of case-control and cohort studies were basically consistent; however, cohort studies were more inclined to a positive association between dietary fat intake and ovarian cancer risk, though no statistical significance was obtained. The summary results were modified by menopausal status, hormone replacement therapy, BMI, and pregnancy times. (Table 4-1 and Table 4-2.)

Sensitivity analysis showed that the results obtained for the association between saturated fat intake and ovarian cancer risk were significantly influenced by one study [29], which didn't adjust hormone use and pregnancy times.

**Table 4-1:** Subgroup analysis based on study characteristics

	Total fat		Animal fat		Plant fat	
	*N*	RR (95%CI)	*N*	RR (95%CI)	*N*	RR (95%CI)
All studies	17	1.19(1.04,1.37) I^2^=60.1%, p=0.001	10	1.21(0.99,1.47) I^2^=68.6%, p=0.001	9	0.95(0.83,1.09) I^2^=29.2%, p=0.185
Study type						
Case-control	11	1.32(1.06,1.63) I^2^=69.8%, p=0.000	5	1.50(0.89,2.53) I^2^=82.3%, p=0.000	4	0.96(0.81,1.12) I^2^=0.0%, p=0.858
Cohort	6	1.10(0.97,1.24) I^2^=4.2%, p=0.389	5	1.09(0.93,1.28) I^2^=28.4%, p=0.232	5	0.93(0.74,1.17) I^2^=61.7%, p=0.034
Geographic location or country						
America	7	1.08(0.95,1.22) I^2^=2.7%, p=0.399	5	1.13(0.96,1.34) I^2^=27.9%, p=0.235	4	0,97(0.85,1.11) I^2^=0.0%, p=0.805
Europe	4	1.25(0.91.1.73) I^2^=83.5%, P=0.000	2	1.08(0.81,1.44) I^2^=58.2%, p=0.012	2	0.90(0.48,1.69) I^2^=89.2%, p=0.002
Others	6	1.33(0.98,1.82) I^2^=63.1%, p=0.019	3	1.69(0.59,4.83) I^2^=88.1%, p=0.000	3	0.88(0.62,1.09) I^2^=0.0%, p=0.796
Adjusted for						
Total energy						
Yes	12	1.12(0.97,1.29) I^2^=48.8%, p=0.034	5	1.07(0.87,1.31) I^2^=48.9%, p=0.098	5	0.90(0.70,1.16) I^2^=63.2%, p=0.028
No	5	1.39(0.99,1.95) I^2^=62.3%, p=0.001	5	1.55(1.00,2.39) I^2^=80.1%, p=0.000	4	0.97(0.83,1.13) I^2^=0.0%, p=0.936
Family history						
Yes	5	1.16 (0.93,1.44) I^2^=44.7%, p=0.143	4	1.17(0.97,1.40) I^2^=28.0%, p=0.244	4	0.96(0.84,1.10) I^2^=29.2%.p=0.185
No	12	1.20(1.00,1.43) I^2^=67.8%, p=0.000	6	1.27(0.89,1.82) I^2^=79.3%, p=0.000	5	0.93(0.71,1.22) I^2^=59.4%, p=0.043
OC use						
Yes	12	1.28(1.09,1.51) I^2^=65.9%, p=0.001	6	1.11(0.97,1.29) I^2^=37.1%, p=0.159	6	0.96(0.82,1.14) I^2^=49.2%, p=0.080
No	5	0.98(0.79,1.22) I^2^=31.5%, p=0.211	4	1.49(0.69,3.21) I^2^=84.2%, p=0.000	3	0.85(0.61,1.17) I^2^=0.0%, p=0.716
BMI						
Yes	6	1.57(1.24,1.99) I^2^=56.3%, p=0.043	3	2.08(1.04,4.14) I^2^=81.5%, p=0.004	2	1.00(0.81,1.25) I^2^=29.2%, p=0.185
No	11	1.04(0.94,1.16) I^2^=17.3%, p=0.284	7	1.03(0.90,1.18) I^2^=22.0%, p=0.261	7	0.92(0.77,1.10) I^2^=46.5%, p=0.082
Menopausal status						
Yes	9	1.20(0.95,1.51) I^2^=75.2%, p=0.000	4	1.29(0.80,2.08) I^2^=82.5%, p=0.001	4	1.08(0.90,1.30) I^2^=8.4%, p=0.351
No	8	1.17(0.99,1.38) I^2^=38.7%, p=0.121	6	1.21(0.98,1.49) I^2^=50.0%, p=0.075	5	0.89(0.76,1.05) I^2^=25.7%, p=0.185
Hormone use						
Yes	3	1.08(0.85,1.37) I^2^=41.7%, p=0.180	2	1.15(0.85,1.55) I^2^=48.3%,0.164	2	0.99(0.81,1.21) I^2^=0.0%, p=0.982
No	14	1.23(1.04,1.45) I^2^=66.6%, p=0.000	8	1.25(0.97,1.62) I^2^=73.4%, p=0.000	7	0.92(0.76,1.11) I^2^=46.7%, p=0.081
Pregnancy times						
Yes	9	1.10(0.98,1.25) I^2^=15.7%, p=0.303	6	1.05(0.8,1.23) I^2^=33.5%, p=0.185	6	0.90(0.73,1.11) I^2^=0.0%, p=0.990
No	8	1.33(1.01,1.74) I^2^=78.2%, p=0.000	4	1.64(1.00,2.69) I^2^=80.6%, p=0.001	3	1.00(0.83,1.21) I^2^=55.4%, p=0.047

**Table 4-2:** Subgroup analysis based on study characteristics

	Monounsaturated fat		Polyunsaturated fat		Dairy fat	
	*N*	RR (95%CI)	*N*	RR (95%CI)	*N*	RR (95%CI)
All studies	13	0.98(0.87,1.09) I^2^=42.6%, p=0.052	13	0.97(0.86,1.11) I^2^=48.9%, p=0.024	6	1.05(0.92,1.19) I^2^=16.3%, p=0.309
Study type						
Case-control	8	0.96(0.83,1.12) I^2^=55.2%, p=0.029	8	0.93(0.82,1.05) I^2^=26.7%, p=0.216	1	0.95(0.79,1.14)
Cohort	5	1.04(0.88,1.22) I^2^=4.5, p=0.381	5	1.06(0.86,1.31) I^2^=49.8%, p=0.093	5	1.10(0.94,1.28) I^2^=13.7%, p=0.327
Geographic location/country						
America	6	0.98(0.85,1.13) I^2^=0.0%, p=0.507	6	0.97(0.76,1.25) I^2^=57.9%, p=0.050	4	0.98(0.86,1.10) I^2^=0.0%, p0.937
Europe	4	0.92(0.77,1.10) I^2^=56.4%, p=0.076	4	1.03(0.89,1.19) I^2^=35.3%, p=0.200	2	1.37(1.05,1.79) I^2^=0.0%, p=0.524
Others	3	1.00(0.72,1.38) I^2^=67.1%, p=0.048	3	0.92(0.65,1.32) I^2^=68.8%, p=0.041	0	
Adjust for						
Total energy						
Yes	9	0.98(0.84,1.14) I^2^=47.8%, p=0.063	9	099(0.84,1.17) I^2^=59.2%, p=0.016	4	1.03(0.87,1.23) I^2^=30.4%, p=0.230
No	4	0.93(0.79,1.10) I^2^=32.4%, p=0.218	4	0.93(0.80,1.08) I^2^=14.6%, p=0.319	2	1.10(0.88,1.37) I^2^=21.3%, p=0.260
Family history						
Yes	4	1.00(0.84,1.19) I^2^=29.0%, p=0.238	4	0.92(0.73,1.15) I^2^=60.8%, p=0.054	2	0.97(0.84,1.12) I^2^=0.0%, p=0.684
No	9	0.95(0.82,1.11) I^2^=50.6%, p=0.048	9	1.02(0.88,1.18) I^2^=42.9%, p=0.092	4	1.15(0.94,1.41) I^2^=25.1%, p=0.684
OC use						
Yes	8	0.99(0.89,1.10) I^2^=26.4%, p=0.227	8	1.00(0.86,1.15) I^2^=55.9%, p=0.034	5	1.08(0.93,1.25) I^2^=27.3%, p=0.239
No	5	0.90(0.68,1.20) I^2^=61.0%, p=0.036	5	0.93(0.71,1.21) I^2^=53.8%, p=0.070	1	0.94(0.70,1.27)
BMI						
Yes	9	1.20(0.94,1.52) I^2^=0.0%, p=0.709	9	1.27(1.03,1.58) I^2^=0.0%, p=0.984	1	0.94(0.70,1.27)
No	4	0.93(0.83,1.05) I^2^=47.0%, p=0.058	4	0.93(0.82,1.05) I^2^=49.8%, p=0.043	5	1.08(0.93,1.25) I^2^=27.3%, p=0.239
Menopausal status						
Yes	6	1.01(0.83,1.23) I^2^=53.9%p=0.070	5	1.10(0.87,1.39) I^2^=53.3%, p=0.093	3	1.00(0.85,1.18) I^2^=0.0%, p=0.876
No	7	0.93(0.81,1.08) I^2^=38.2%, p=0.138	8	0.92(0.82,1.02) I^2^=16.6%, p=0.299	3	1.17(0.88,1.57) I^2^=62.6%, p=0.069
Hormone use						
Yes	2	1.05(0.79,1.39) I^2^=0.0%, p=0.847	2	1.22(0.95,1.55) I^2^=0.0%, p=0.642	2	0.98(0.78,1.24) I^2^=0.0%, p=0.622
No	11	0.96(0.84,1.09) I^2^=52.0%, p=0.027	11	0.95(0.83,1.08) I^2^=52.3%, p=0.026	4	1.10(0.91,1.32) I^2^=45.5%, p=0.139
Pregnancy times						
Yes	8	1.02(0.89,1.17) I^2^=38.6%, p=0.122	8	0.93(0.78,1.12) I^2^=62.9%, p=0.009	3	1.02(0.88,1.18) I^2^=12.2%, p=0.320
No	5	0.87(0.77,0.98) I^2^=0.0%, p=0.047	5	1.02(0.90,1.17) I^2^=0.0%, p=0.405	3	1.11(0.85,1.46) I^2^=40.6%, p=0.186

*P*-value for heterogeneity within each subgroup.

### Publication bias

We found no evidence of publication bias with regard to dietary fat consumption and ovarian cancer risk by means of visual inspection of funnel plots and formal statistical tests, including Begg rank correlation test and Egger linear regression test (all *P* > 0.05).

## DISCUSSION

Dietary fat, as one of the most controversial dietary factors in nutritional epidemiology, might elevate the incidence of hormone related cancers, including breast, endometrial and ovarian cancers, but discrepant observational results have been reported. We thoroughly searched the literature, and found the incidence of two important cancer types, breast cancer and stomach cancer, were in relation to the high consumption of dietary fat. Breast cancer was traditionally considered to be linked with western lifestyles, other including prostate and colorectal cancers, and the inflammatory bowel diseases; IBD, Crohn's disease (CD). Stomach cancer was considered to be linked more with eastern lifestyle. The included two meta-analyses [7, 8] indicated positive associations between dietary fat intake and breast and stomach cancer. Considering the regional difference of these two cancers, we can conclude that the effect of dietary fat on cancer risk may be independent of the region. To define the effect of dietary fat on ovarian cancer risk, we conducted this meta-analysis to clear this research subject.

The results of this meta-analysis including case-control and cohort studies indicated that consumption of total dietary fat and trans fat increased the risk of ovarian cancer. These findings were consistent with the results in specific epidemical studies in NIH-AARP cohort study [35] and an earlier meta-analysis [11], which reported positive associations between total fat intake and EOC risk. Contrasted with our meta-analysis, a pooled analysis [12], three cohort studies [34, 39, 40] and several case-control studies [20, 24, 27, 29] observed no association between total fat consumption and EOC risk. Consistent with a pooled analysis [12] of 12 cohort studies, we observed no significant associations of consumption of dietary fat and risk of ovarian cancer subtypes. There was also no relevance in the associations with dietary fat intake and the risk of different histological subtypes of EOC in an American case-control study [27] and an Italian case-control study [30]. Limited studies involved in associations between dietary fat intakes and EOC risk attribute to different histological subtypes of EOC. Merritt [34] (EPIC) reported high intake of polyunsaturated fatty acids can increase risk for serous EOC. Blank [35] observed an increased risk of serous EOC by total energy from animal fat and inverse associations with risk of EOC observed for the intakes of plant fat and polyunsaturated FAs. Beral [49] reported that serous subtype appeared to have more consistent global distribution, followed by endometrioid subtype, whereas mucinous and clear cell subtypes varied significantly across countries. Among these four main subtypes, the clear cell subtype was least studied, which has higher prevalence in Asia [50–52] and was more frequently found in younger women [52]. In the current meta-analysis, there was only one study [27] from America involving ovarian clear cell carcinoma (OCCC). In addition, the survival outcome of OCCC was comparable to that of serous EOC in terms of early-stage disease [50, 53–55], but worse with respect to advanced-stage disease [56–62]. Therefore, more studies, including epidemiological and clinical studies, should be carried out in Asia and other “new regions” (Central and South America, Africa) [63].

Subgroup analysis based on the characteristics of included studies suggested that menopausal status, hormone replacement therapy, BMI, and pregnancy times can modify the association between dietary fat intake and ovarian cancer risk. These factors are related to exposure to estrogens [64, 65]. A reanalysis [49] of epidemiological data suggested estrogen monotherapy or estrogen and progesterone combination therapy could elevate the risk of ovarian cancer, specifically serous or endometroid tumors. In ovarian tissues, estrogen receptors are also expressed [66]; the ratios of estrogen-DNA adduct depurination to estrogen metabolites and conjugates in ovarian cancer cases are significantly higher than controls [67]. We speculated that hormonal pathways might play a positive role in the development of ovarian cancer. High consumption of dietary fat could stimulate the secretion of extra ovarian estrogen [68, 69], which can exert tumor-promoting activity *via* mitogenic effects on ERα- positive [70, 71] or negative [72] tumor cells, therefore increasing the risk of ovarian cancer [66]. What's more, obese women may suffer from insulin resistance, and concurrent hyperinsulinemia with excess insulin-like growth factor-1 receptor (IGF-1) could additionally induce androgen steroidogenesis [73] and lead to tumor development [74, 75].

An important highlight of our meta-analysis is that we analyzed the association between dietary fat intake and the risk of ovarian cancer subtypes. We found that serous ovarian cancer incidence was more susceptible to dietary fat intake. However, these results should be interpreted with caution. The insufficient number of included cases and potential misclassification of pathological subtypes may contribute to the statistical difference observed.

Several limitations of this meta-analysis should be considered. First, there was substantial heterogeneity across studies assessing the associations of dietary fat intake with ovarian cancer risk. Considering the varieties of the characteristics of included populations, and study designs and types, the existence of substantial heterogeneity was reasonable, and we conducted subgroup analysis to reduce its effect on the results. Second, misclassification bias, which stemmed mainly from the misclassification of dietary assessments and pathological subtypes of ovarian cancer, should be paid enough attention to. Misclassification of dietary assessments may result from the differences across nutrient databases or designed questionnaires. Diagnosis, pathology review, and classification methods could cause misclassification bias of pathological subtypes of ovarian cancer. Third, we couldn't rule out the effects of confounding factors and various statistical biases on our results. Furthermore, controls and confounding factor adjustment methods across individual studies were not consistent. With more and more basic and clinical researches in recent years, and the increasing understanding of the relationship between diet and health, the confounding factors controlled have markedly increased in number, and bias was inevitable. Forth, although no publication bias was found, its possible effect cannot be totally excluded.

In conclusion, the present meta-analysis of case-control and cohort studies indicate that increased consumption of total fat, saturated fat and trans-fat may be associated with an increased risk of ovarian cancer. Among the dietary fats, saturated fats can significantly increase serous and endometroid ovarian cancer, with the risk of serous ovarian cancer more susceptible to dietary fat intake. In addition, subgroup analysis data suggested that menopausal status, hormone replacement therapy, BMI, and pregnancy times may serve as potential effect modifiers. Future studies should focus more on specific pathological subtypes of ovarian cancer as well as the influence of molecular mechanisms and genetic factors on the association of dietary fat and ovarian cancer.

## MATERIALS AND METHODS

### Search strategy

We obtained the literature published in any language to December 2015 by fully searching the PubMed database. The search terms used were “diet”, “dietary fat” in combination with “ovarian cancer,” “ovarian neoplasm” or “ovarian carcinoma”, without restrictions. In addition, we reviewed the reference lists of retrieved studies and recent reviews to supplement electronic database searches.

### Study selection

Study selection included initial screening of titles or abstracts, and a second one for full texts. Studies were eligible for inclusion if they met the following criteria: 1) observational studies which enrolled patients with proven epithelial ovarian cancers excluding tumors of “borderline malignant potential” histopathology, 2) patients enrolled were adults (≥18 yr of age), 3) studies containing available data showing association(s) between intake of dietary fat (total, saturated, animal, dairy or unsaturated fat) and ovarian cancer, and 4) odds ratio or relative risk (RR) with 95% confidence interval (CI) for each variable or availability of raw data to calculate these parameters.

### Data extraction

All data were extracted with a data-collection form. Information was recorded as follows: last name of the first author, publication year, study population, period, country, sample size; risk estimate from multivariable model for the highest *versus* lowest category of dietary fat intake with the corresponding 95% CI; statistical adjustment for the main confounding factors of interest.

Data extraction and study selection were performed by 3 authors (Qiu WL, Lu H and Qi YN) independently. Any disagreements were resolved by discussion.

### Statistical methods

The association between dietary fat consumption and the risk of ovarian cancer was our main analytical object. Dietary fats in this meta-analysis were defined as total fat, animal fat, plant fat, dairy fat, saturated fat, monounsaturated fat, polyunsaturated fat and trans-fat. Relative risk (RR) was used as the common measure of association in this meta-analysis, and the random-effects model was selected to calculate summary RRs and 95% CIs associated with dietary fats. Q statistic (significance level at *P* < 0.10) and I^2^ statistic, a quantitative measure of inconsistency across studies [13], were applied for heterogeneity assessment of RRs across studies. Subgroup analyses stratified by geographic region (country), study type, and study characteristics were carried out to investigate potential sources of heterogeneity. Sensitivity analyses were performed by excluding one study at a time to assess the influence of a single study on the overall risk estimate. Publication bias was assessed with funnel plots, Egger's test [14], and Begg's test [15] (all *P* > 0.05). The Stata version 12.0 software (StataCorp) was used for statistical analyses.

## Abbreviations

EOC: epithelial ovarian cancer
RR: relative risk
OR: odds ratio
CI: confidential interval
BMI: body mass index.

## References

[1] Permuth-Wey J, Sellers TA (2009). Epidemiology of ovarian cancer. Methods Mol Biol.

[2] Jemal A, Bray F, Center MM, Ferlay J, Ward E, Forman D (2011). Global cancer statistics. CA Cancer J Clin.

[3] Allen TW (1991). Guide to clinical preventive services. Report of the US Preventive Services Task Force. J Am Osteopath Assoc.

[4] Siegel R, Naishadham D, Jemal A (2012). Cancer statistics, 2012. CA Cancer J Clin.

[5] Armstrong B, Doll R (1975). Environmental factors and cancer incidence and mortality in different countries, with special reference to dietary practices. Int J Cancer.

[6] Parkin DM, Bray F, Ferlay J, Pisani P (2005). Global cancer statistics, 2002. CA Cancer J Clin.

[7] Cao Y, Hou L, Wang W (2016). Dietary total fat and fatty acids intake, serum fatty acids and risk of breast cancer: A meta-analysis of prospective cohort studies. Int J Cancer.

[8] Han J, Jiang Y, Liu X, Meng Q, Xi Q, Zhuang Q, Han Y, Gao Y, Ding Q, Wu G (2015). Dietary Fat Intake and Risk of Gastric Cancer: A Meta-Analysis of Observational Studies. PLoS One.

[9] Prentice RL, Sheppard L (1990). Dietary fat and cancer: consistency of the epidemiologic data, and disease prevention that may follow from a practical reduction in fat consumption. Cancer Causes Control.

[10] Hou R, Wu QJ, Gong TT, Jiang L (2015). Dietary fat and fatty acid intake and epithelial ovarian cancer risk: evidence from epidemiological studies. Oncotarget.

[11] Huncharek M, Kupelnick B (2001). Dietary fat intake and risk of epithelial ovarian cancer: a meta-analysis of 6,689 subjects from 8 observational studies. Nutr Cancer.

[12] Genkinger JM, Hunter DJ, Spiegelman D, Anderson KE, Beeson WL, Buring JE, Colditz GA, Fraser GE, Freudenheim JL, Goldbohm RA, Hankinson SE, Koenig KL, Larsson SC (2006). A pooled analysis of 12 cohort studies of dietary fat, cholesterol and egg intake and ovarian cancer. Cancer Causes Control.

[13] Higgins JP, Thompson SG, Deeks JJ, Altman DG (2003). Measuring inconsistency in meta-analyses. BMJ.

[14] Egger M, Davey SG, Schneider M, Minder C (1997). Bias in meta-analysis detected by a simple, graphical test. BMJ.

[15] Begg CB, Mazumdar M (1994). Operating characteristics of a rank correlation test for publication bias. Biometrics.

[16] Cramer DW, Welch WR, Hutchison GB, Willett W, Scully RE (1984). Dietary animal fat in relation to ovarian cancer risk. Obstet Gynecol.

[17] Risch HA, Jain M, Marrett LD, Howe GR (1994). Dietary fat intake and risk of epithelial ovarian cancer. J Natl Cancer Inst.

[18] Risch HA, Marrett LD, Jain M, Howe GR (1996). Differences in risk factors for epithelial ovarian cancer by histologic type. Results of a case-control study. Am J Epidemiol.

[19] Shu XO, Gao YT, Yuan JM, Ziegler RG, Brinton LA (1989). Dietary factors and epithelial ovarian cancer. Br J Cancer.

[20] Slattery ML, Schuman KL, West DW, French TK, Robison LM (1989). Nutrient intake and ovarian cancer. Am J Epidemiol.

[21] Tzonou A, Hsieh CC, Polychronopoulou A, Kaprinis G, Toupadaki N, Trichopoulou A, Karakatsani A, Trichopoulos D (1993). Diet and ovarian cancer: a case-control study in Greece. Int J Cancer.

[22] La Vecchia C, Decarli A, Negri E, Parazzini F, Gentile A, Cecchetti G, Fasoli M, Franceschi S (1987). Dietary factors and the risk of epithelial ovarian cancer. J Natl Cancer Inst.

[23] Webb PM, Bain CJ, Purdie DM, Harvey PW, Green A (1998). Milk consumption, galactose metabolism and ovarian cancer (Australia). Cancer Causes Control.

[24] Pan SY, Ugnat AM, Mao Y, Wen SW, Johnson KC (2004). A case-control study of diet and the risk of ovarian cancer. Cancer Epidemiol Biomarkers Prev.

[25] Zhang M, Yang ZY, Binns CW, Lee AH (2002). Diet and ovarian cancer risk: a case-control study in China. Br J Cancer.

[26] McCann SE, Freudenheim JL, Marshall JR, Graham S (2003). Risk of human ovarian cancer is related to dietary intake of selected nutrients, phytochemicals and food groups. J Nutr.

[27] Merritt MA, Cramer DW, Missmer SA, Vitonis AF, Titus LJ, Terry KL (2014). Dietary fat intake and risk of epithelial ovarian cancer by tumour histology. Br J Cancer.

[28] Zhang M, Lee AH, Binns CW (2004). Reproductive and dietary risk factors for epithelial ovarian cancer in China. Gynecol Oncol.

[29] Salazar-Martinez E, Lazcano-Ponce EC, Gonzalez LG, Escudero-De lRP, Hernandez-Avila M (2002). Nutritional determinants of epithelial ovarian cancer risk: a case-control study in Mexico. Oncology.

[30] Chiaffarino F, Parazzini F, Bosetti C, Franceschi S, Talamini R, Canzonieri V, Montella M, Ramazzotti V, Franceschi S, La Vecchia C (2007). Risk factors for ovarian cancer histotypes. Eur J Cancer.

[31] Hu J, La Vecchia C, de Groh M, Negri E, Morrison H, Mery L (2011). Dietary transfatty acids and cancer risk. Eur J Cancer Prev.

[32] Merritt MA, Poole EM, Hankinson SE, Willett WC, Tworoger SS (2014). Dairy food and nutrient intake in different life periods in relation to risk of ovarian cancer. Cancer Causes Control.

[33] Bertone ER, Rosner BA, Hunter DJ, Stampfer MJ, Speizer FE, Colditz GA, Willett WC, Hankinson SE (2002). Dietary fat intake and ovarian cancer in a cohort of US women. Am J Epidemiol.

[34] Merritt MA, Riboli E, Weiderpass E, Tsilidis KK, Overvad K, Tjonneland A, Hansen L, Dossus L, Fagherazzi G, Baglietto L, Fortner RT, Ose J, Steffen A (2014). Dietary fat intake and risk of epithelial ovarian cancer in the European Prospective Investigation into Cancer and Nutrition. Cancer Epidemiol.

[35] Blank MM, Wentzensen N, Murphy MA, Hollenbeck A, Park Y (2012). Dietary fat intake and risk of ovarian cancer in the NIH-AARP Diet and Health Study. Br J Cancer.

[36] Kiani F, Knutsen S, Singh P, Ursin G, Fraser G (2006). Dietary risk factors for ovarian cancer: the Adventist Health Study (United States). Cancer Causes Control.

[37] Mommers M, Schouten LJ, Goldbohm RA, van den Brandt PA (2006). Dairy consumption and ovarian cancer risk in the Netherlands Cohort Study on Diet and Cancer. Br J Cancer.

[38] Kushi LH, Mink PJ, Folsom AR, Anderson KE, Zheng W, Lazovich D, Sellers TA (1999). Prospective study of diet and ovarian cancer. Am J Epidemiol.

[39] Chang ET, Lee VS, Canchola AJ, Clarke CA, Purdie DM, Reynolds P, Anton-Culver H, Bernstein L, Deapen D, Peel D, Pinder R, Ross RK, Stram DO (2007). Diet and risk of ovarian cancer in the California Teachers Study cohort. Am J Epidemiol.

[40] Gilsing AM, Weijenberg MP, Goldbohm RA, van den Brandt PA, Schouten LJ (2011). Consumption of dietary fat and meat and risk of ovarian cancer in the Netherlands Cohort Study. Am J Clin Nutr.

[41] Mori M, Harabuchi I, Miyake H, Casagrande JT, Henderson BE, Ross RK (1988). Reproductive, genetic, and dietary risk factors for ovarian cancer. Am J Epidemiol.

[42] Mori M, Miyake H (1988). Dietary and other risk factors of ovarian cancer among elderly women. Jpn J Cancer Res.

[43] Engle A, Muscat JE, Harris RE (1991). Nutritional risk factors and ovarian cancer. Nutr Cancer.

[44] Tomao S, Taggi F, Sberna RC, Villani C (1992). Ovarian cancer and dietary habits. Eur J Gynaecol Oncol.

[45] Bosetti C, Negri E, Franceschi S, Pelucchi C, Talamini R, Montella M, Conti E, La Vecchia C (2001). Diet and ovarian cancer risk: a case-control study in Italy. Int J Cancer.

[46] Pirozzo S, Purdie D, Kuiper-Linley M, Webb P, Harvey P, Green A, Bain C (2002). Ovarian cancer, cholesterol, and eggs: a case-control analysis. Cancer Epidemiol Biomarkers Prev.

[47] Parazzini F, Chiaffarino F, Negri E, Surace M, Benzi G, Franceschi S, Fedele L, La Vecchia C (2004). Risk factors for different histological types of ovarian cancer. Int J Gynecol Cancer.

[48] Lubin F, Chetrit A, Modan B, Freedman LS (2006). Dietary intake changes and their association with ovarian cancer risk. J Nutr.

[49] Beral V, Gaitskell K, Hermon C, Moser K, Reeves G, Peto R (2015). Menopausal hormone use and ovarian cancer risk: individual participant meta-analysis of 52 epidemiological studies. Lancet.

[50] Chan JK, Teoh D, Hu JM, Shin JY, Osann K, Kapp DS (2008). Do clear cell ovarian carcinomas have poorer prognosis compared to other epithelial cell types? A study of 1411 clear cell ovarian cancers. Gynecol Oncol.

[51] Orezzoli JP, Russell AH, Oliva E, Del CMG, Eichhorn J, Fuller AF (2008). Prognostic implication of endometriosis in clear cell carcinoma of the ovary. Gynecol Oncol.

[52] Tay SK, Cheong MA (2014). Evidence for ethnic and environmental contributions to frequency of ovarian clear cell carcinoma. Aust N Z J Obstet Gynaecol.

[53] Lee YY, Kim TJ, Kim MJ, Kim HJ, Song T, Kim MK, Choi CH, Lee JW, Bae DS, Kim BG (2011). Prognosis of ovarian clear cell carcinoma compared to other histological subtypes: a meta-analysis. Gynecol Oncol.

[54] Higashi M, Kajiyama H, Shibata K, Mizuno M, Mizuno K, Hosono S, Kawai M, Nakanishi T, Nagasaka T, Kikkawa F (2011). Survival impact of capsule rupture in stage I clear cell carcinoma of the ovary in comparison with other histological types. Gynecol Oncol.

[55] Suh DH, Park JY, Lee JY, Kim BG, Lim MC, Kim JW, Bae DS, Park SY, Nam JH, Kim K, No JH, Kim YB (2015). The clinical value of surgeons' efforts of preventing intraoperative tumor rupture in stage I clear cell carcinoma of the ovary: A Korean multicenter study. Gynecol Oncol.

[56] Takano M, Kikuchi Y, Yaegashi N, Kuzuya K, Ueki M, Tsuda H, Suzuki M, Kigawa J, Takeuchi S, Tsuda H, Moriya T, Sugiyama T (2006). Clear cell carcinoma of the ovary: a retrospective multicentre experience of 254 patients with complete surgical staging. Br J Cancer.

[57] Ye S, Yang J, You Y, Cao D, Bai H, Lang J, Chen J, Shen K (2014). Comparative study of ovarian clear cell carcinoma with and without endometriosis in People's Republic of China. Fertil Steril.

[58] Shevchuk MM, Winkler-Monsanto B, Fenoglio CM, Richart RM (1981). Clear cell carcinoma of the ovary: a clinicopathologic study with review of the literature. Cancer.

[59] Jenison EL, Montag AG, Griffiths CT, Welch WR, Lavin PT, Greer J, Knapp RC (1989). Clear cell adenocarcinoma of the ovary: a clinical analysis and comparison with serous carcinoma. Gynecol Oncol.

[60] Goff BA, de la Cuesta R S, Muntz HG, Fleischhacker D, Ek M, Rice LW, Nikrui N, Tamimi HK, Cain JM, Greer BE, Fuller AF (1996). Clear cell carcinoma of the ovary: a distinct histologic type with poor prognosis and resistance to platinum-based chemotherapy in stage III disease. Gynecol Oncol.

[61] Sugiyama T, Kamura T, Kigawa J, Terakawa N, Kikuchi Y, Kita T, Suzuki M, Sato I, Taguchi K (2000). Clinical characteristics of clear cell carcinoma of the ovary: a distinct histologic type with poor prognosis and resistance to platinum-based chemotherapy. Cancer.

[62] Makar AP, Baekelandt M, Trope CG, Kristensen GB (1995). The prognostic significance of residual disease, FIGO substage, tumor histology, and grade in patients with FIGO stage III ovarian cancer. Gynecol Oncol.

[63] Shih WJ (2001). Clinical trials for drug registrations in Asian-Pacific countries: proposal for a new paradigm from a statistical perspective. Control Clin Trials.

[64] Hunn J, Rodriguez GC (2012). Ovarian cancer: etiology, risk factors, and epidemiology. Clin Obstet Gynecol.

[65] Salehi F, Dunfield L, Phillips KP, Krewski D, Vanderhyden BC (2008). Risk factors for ovarian cancer: an overview with emphasis on hormonal factors. J Toxicol Environ Health B Crit Rev.

[66] Lukanova A, Kaaks R (2005). Endogenous hormones and ovarian cancer: epidemiology and current hypotheses. Cancer Epidemiol Biomarkers Prev.

[67] Zahid M, Beseler CL, Hall JB, LeVan T, Cavalieri EL, Rogan EG (2014). Unbalanced estrogen metabolism in ovarian cancer. Int J Cancer.

[68] Hill MJ, Goddard P, Williams RE (1971). Gut bacteria and aetiology of cancer of the breast. Lancet.

[69] Wu AH, Pike MC, Stram DO (1999). Meta-analysis: dietary fat intake, serum estrogen levels, and the risk of breast cancer. J Natl Cancer Inst.

[70] Gallo D, De Stefano I, Grazia PM, Scambia G, Ferrandina G (2012). Estrogen receptor beta in cancer: an attractive target for therapy. Curr Pharm Des.

[71] Ribeiro JR, Freiman RN (2014). Estrogen signaling crosstalk: Implications for endocrine resistance in ovarian cancer. J Steroid Biochem Mol Biol.

[72] Ciucci A, Zannoni GF, Buttarelli M, Lisi L, Travaglia D, Martinelli E, Scambia G, Gallo D (2016). Multiple direct and indirect mechanisms drive estrogen-induced tumor growth in high grade serous ovarian cancers. Oncotarget.

[73] Kaaks R, Lukanova A (2001). Energy balance and cancer: the role of insulin and insulin-like growth factor-I. Proc Nutr Soc.

[74] Khandwala HM, McCutcheon IE, Flyvbjerg A, Friend KE (2000). The effects of insulin-like growth factors on tumorigenesis and neoplastic growth. Endocr Rev.

[75] Becker S, Dossus L, Kaaks R (2009). Obesity related hyperinsulinaemia and hyperglycaemia and cancer development. Arch Physiol Biochem.

